# Granulocytes and Vascularization Regulate Uterine Bleeding and Tissue Remodeling in a Mouse Menstruation Model

**DOI:** 10.1371/journal.pone.0041800

**Published:** 2012-08-07

**Authors:** Astrid Menning, Alexander Walter, Marion Rudolph, Isabella Gashaw, Karl-Heinrich Fritzemeier, Lars Roese

**Affiliations:** Therapeutic Research Group Oncology/Gynecological Therapy, Bayer HealthCare Pharmaceuticals, Berlin, Germany; Konkuk University, Republic of Korea

## Abstract

Menstruation-associated disorders negatively interfere with the quality of life of many women. However, mechanisms underlying pathogenesis of menstrual disorders remain poorly investigated up to date. Among others, this is based on a lack of appropriate pre-clinical animal models. We here employ a mouse menstruation model induced by priming mice with gonadal hormones and application of a physical stimulus into the uterus followed by progesterone removal. As in women, these events are accompanied by menstrual-like bleeding and tissue remodeling processes, i.e. disintegration of decidualized endometrium, as well as subsequent repair. We demonstrate that the onset of bleeding coincides with strong upregulation of inflammatory mediators and massive granulocyte influx into the uterus. Uterine granulocytes play a central role in regulating local tissue remodeling since depletion of these cells results in dysregulated expression of matrix modifying enzymes. As described here for the first time, uterine blood loss can be quantified by help of tampon-like cotton pads. Using this novel technique, we reveal that blood loss is strongly reduced upon inhibition of endometrial vascularization and thus, is a key regulator of menstrual bleeding. Taken together, we here identify angiogenesis and infiltrating granulocytes as critical determinants of uterine bleeding and tissue remodeling in a mouse menstruation model. Importantly, our study provides a technical and scientific basis allowing quantification of uterine blood loss in mice and thus, assessment of therapeutic intervention, proving great potential for future use in basic research and drug discovery.

## Introduction

During a women’s reproductive phase the endometrium undergoes repetitive cycles of proliferation, differentiation, breakdown and repair preparing the uterus for implantation and growth of an embryo. If implantation does not occur, the superficial layer of the endometrium (functionalis) is decomposed and shed resulting in menstrual bleeding. The critical event initiating menstruation is the cessation of progesterone (P4) production due to regression of the corpus luteum in the absence of pregnancy. In response to declining P4 levels, an array of inflammatory, vasoactive and hypoxic mediators is locally released leading to the induction/activation of extracellular matrix modifying enzymes. As a result, coordinated inflammation, tissue injury and shedding of the functionalis take place being followed by complete, scarless repair and regeneration from the basal layer [1]–[3].

These repetitive cycles of tissue remodeling are highly controlled and disturbances are supposed to result in menstrual disorders such as prolonged and/or excessive blood loss (heavy menstrual bleeding/HMB, menorrhagia) [1], [2]. HMB is defined as a blood loss exceeding 80 ml per cycle, which significantly interferes with a woman’s physical, social, emotional and sometimes even material quality of life [4]. It affects about 9–14% of otherwise healthy, pre-menopausal women and is among the most common reasons for referral to a gynecologist and hysterectomy. Hence, HMB is associated with great socio-economic impact raising a high need to develop appropriate medication [5].

Development of effective therapy against HMB on the one hand is hampered by a poor knowledge on cellular and molecular mechanisms of menstruation and pathogenesis of associated disorders. On the other hand, there is a lack of suitable pre-clinical animal models. This relies on the fact that apart from women, menstruation uniquely occurs in Old World monkeys, the elephant shrew and a few bat species, all sharing the phenomenon of spontaneous decidualization. In the latter species, as a result of a post-ovulatory rise in progesterone levels, endometrial stromal cells extensively proliferate and differentiate into large, polyploid cells destined to support implantation. In the absence of a blastocyst the decidualized tissue is shed as menstrual discharge. In most species, including rodents, decidualization only occurs if a blastocyst penetrates the endometrium. Hence, spontaneous decidualization and menstruation are missing.

Despite these difficulties, mouse models of menstruation have been established [6]–[9]. In these models decidualization is achieved by injection of oil into the uterine lumen of hormonally pre-sensitized mice. One of these models relies on priming ovariectomized mice with estrogen and progesterone [6]–[8]. In response to decidualization and removal of progesterone, endometrial tissue injury, shedding and repair can be observed and were found to share many features of human menstruation. A further approach to sensitize mice for decidualization is based on mating intact female mice with vasectomized males, thereby causing hormone levels similar to those during pregnancy (pseudopregnancy). As recently reported, coinciding with a drop of progesterone, decidualized tissue is shed and overt menstrual-like bleeding takes place [9]. However, in spite of these advances, mechanisms underlying menstruation remain largely elusive and appropriate techniques allowing characterization and quantification of clinical parameters such as uterine blood loss have not been described so far.

In the current study, we show that overt uterine bleeding is induced in the mouse P4 withdrawal model and that it can be quantified in terms of total blood loss as well as bleeding intensity. Our data identify endometrial vascularization and granulocytes as key regulators of menstrual bleeding and local tissue remodeling. Notably, we provide a scientific and technical platform that allows investigation of menstrual disorders and therapeutic intervention in the future.

## Methods

### Mice

Female C57Bl/6N mice were obtained from Charles River WIGA GmbH (Sulzfeld, Germany) and were used at 8–10 weeks of age. All animal experiments were performed under standardized conditions and in accordance with institutional, state and federal guidelines. The study was approved by the Regional Office for Health and Social Affairs in Berlin (LAGeSo; protocol A0384/09.

### Mouse Model of Menstruation and Treatment Regimens

**Figure 1:**
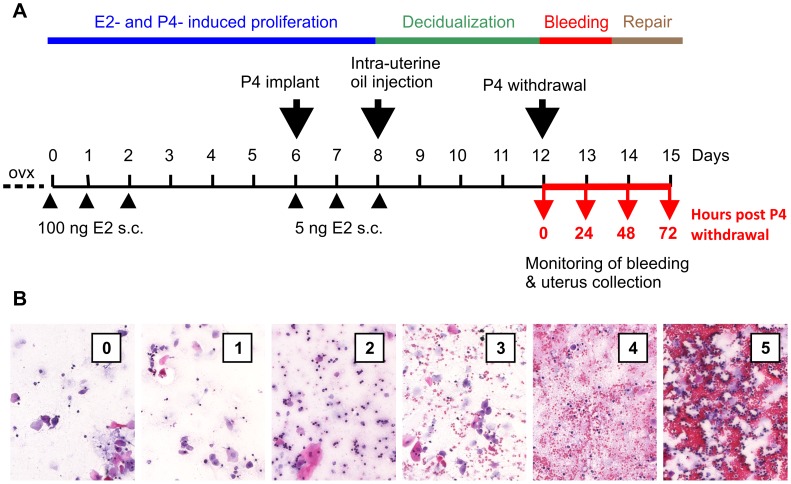
A mouse model of induced menstruation. A: Ovariectomized (ovx) mice were treated with estradiol (E2) injections and progesterone (P4) releasing implants to stimulate endometrial proliferation and differentiation. Decidualization was induced by injection of oil into one uterus horn. Endometrial bleeding in response to removal of the P4 implant was monitored by vaginal smears or blood collection with tampon-like cotton pads. Uteri collected at indicated points of time were used for histology and analysis of gene expression, leukocyte content and enzymatic activity, respectively. B: Cotton swabs were used to take vaginal smears from mice. Based on a scoring system bleeding intensity was evaluated according to erythrocyte density in H/E stained vaginal smears.

One week after ovariectomy female C57Bl/6 mice received s.c. injections of 100 ng 17â-estradiol (E2, internal source) in ethanol/arachis oil (1∶9) on three consecutive days (Figure 1A). After a three days break a progesterone (P4) releasing silastic tube (0.5 mg P4/d, internal source, [10]) was implanted s.c. into the back of mice followed by further applications of 5 ng E2 on three consecutive days. Concomitant with the last E2 treatment 50 µl sesame oil were injected into one uterus horn to induce decidualization. 4 days later the P4 implant was removed to initiate P4 withdrawal. Mice were sacrificed at indicated points of time and uteri were weighed and harvested for further analyses. All surgeries were performed under isofluran-induced anesthesia.

For depletion experiments 150 µg monoclonal rat-anti-mouse Ly-6G(GR-1)-antibody (RB6-8C5; eBioscience, San Diego, CA, USA) in PBS per mouse was administered i.p. once daily (d11–d14). Control mice received ratIgG (Jackson Immunoresearch, Mill Valley, CA, USA).

Inhibition of angiogenesis *in vivo* was performed by s.c. application of Cediranib (AZD2171, AstraZeneca, London, UK) dissolved in Transcutol/Chremophor/H_2_O (1/1/8) to mice at a dose of 10 mg/kg daily (d8–d15). Controls were treated with vehicle alone.

### Assessment of Uterine Blood Loss and Bleeding Intensity

To assess bleeding intensity, cotton swabs soaked with 30 µl PBS solution were used to catch up vaginal fluid containing uterine blood. Swabs were pressed onto a glass slide. Dried smears were incubated in 70% ethanol for 2 hours and hematoxylin/eosin (H&E) stained using an automatic Leica Multistainer and Coverslipper (Leica ST5020+CV5030, Leica Microsystems, Wetzlar, Germany). Using a scoring system bleeding intensity was evaluated in vaginal smears by microscopy (Figure 1B): 0 =  no erythrocytes; 1 =  few erythrocytes; 2 =  evenly scattered erythrocytes; 3 =  frequent occurrence of erythrocytes with little cluster formation; 4 =  very frequent occurrence of erythrocytes, strong cluster formation; 5 =  massive accumulations and clustering of erythrocytes.

To quantify the total amount of blood loss, tampon-like cotton pads (4–4.8 mm diameter, Roeko, Coltène/Whaledent, Altstätten, Switzerland) were inserted into the vagina of mice at P4 withdrawal. Mice additionally received a paper collar to prevent removal of tampons. Tampons were changed twice daily and collected for each mouse separately. Blood volume was quantified by the alkaline hematine method reported elsewhere [11]. Briefly, tampons were left to dry at room temperature. Heme chromogens were dissolved in 1000 µl 5% NaOH (w/v) and rotated over night at room temperature. Optical density of the eluate was measured in an ELISA Reader at a wavelength of 546 nm. Blood volume contained in cotton swabs was calculated based on a regression curve of standards prepared from venous blood.

### Histology and Immunohistochemistry

Cross sections of formalin(3.7%)-fixed, paraffin-embedded uterine tissue was deparaffinized, hydrated and H&E stained using an automatic Leica Multistainer and Coverslipper (Leica ST5020+CV 5030, Leica Microsystems, Wetzlar, Germany).

For detection of CD31/PECAM expressing endothelium, anti-mouse-CD31-IgG (sc-1506-R, rabbit polyclonal, Santa Cruz Biochemicals, Santa Cruz, CA, USA) was used as primary antibody. The Dako Envision+ System-HRP (Dako, Glostrup, Denmark) and NovaRED substrate solution (Vector Laboratories, Burlingame, CA, USA) was applied to develop specific staining. Slides were additionally counter-stained with H&E.

### Isolation and Flow Cytometric Analysis of Uterine Leukocytes

Half of one decidualized uterus horn per mouse was used for FACS analysis. Uterus horns were cut longitudinally and transversally into pieces of 2 mm and were agitated (250 rpm) for 45 min at 37°C in 2 ml digestion medium (RPMI1640 supplemented with 5% FCS, 100 U/ml Penicillin, 0.1 mg/ml Streptomycin, 0.1 U/ml Collagenase D (Roche, Mannheim, Germany), 200 U/ml Collagenase VIII (Sigma-Aldrich, St. Louis, MO, USA). Cells were washed twice with excess cold PBS+2%FCS. Cells were resuspended in PBS+2%FCS and stained with indicated antibodies for 15 min at 4°C. To exclude dead cells, Dapi (4′,6-Diamidino-2-phenyl-indole dihydrochloride, Sigma-Aldrich, St. Louis, MO, USA) in appropriate dilutions was added to samples. Flow cytometry was performed using the FACS Canto II Flow Cytometer and FACS Diva software (BD Biosciences, San Diego, CA, USA).

### Antibodies and Reagents

The following antibodies were used in this study: anti-mouse-CD45-APC-eFluor780 (30-F11), anti-mouse-Ly-6G(Gr-1)-eFluor450 (RB6-8C5), anti–mouse-CD4-FITC (GK1.5), anti-mouse-CD8-PE-Cy5 (H35-17.2), anti–mouse-CD19-eFluor450 (1D3), anti-mouse-F4/80-FITC (BM8), anti-mouse-CD335(NKp46)-PerCP-eFluor710 (29A1.4), anti-mouse-CD11b-PE-Cy7 (M1/70) (all purchased from eBioscience, San Diego, CA, USA) and anti-mouse-LFA-1á-PE (M17/4, BD Biosciences, San Diego, CA, USA).

### Gene Expression Analysis by RT PCR

Uterine tissue was homogenized in RLT lysis buffer (Qiagen, Hilden, Germany) using the Precellys24 homogenizer (1.4 mm Ceramic beads; 2×6000 rpm; Peqlab, Erlangen, Germany) and total-RNA was isolated using the QIAsymphony RNA kit on the QIAsymphony SP robot for automated sample preparation (Qiagen). Reverse transcription of 1 µg total-RNA was performed using the SuperScript III-first strand synthesis system (Invitrogen, Carlsbad, CA, USA) according to the Random Hexamer procedure. Gene expression analysis was performed with 25 ng cDNA per reaction on the SDS7900HT Real-Time PCR System using TaqMan-Assays (Applied Biosystems, Carlsbad, CA, USA) and Fast Blue qPCR Mastermix Plus (Eurogentec, Seraing, Liège, Belgium). For relative quantification, Cyclophilin A was used as an endogenous control. Relative expression levels were calculated according to the comparative ▵▵C_T_-Method.

### Statistics

Data are presented as mean ± standard deviation (SD) or as median as indicated in figure legends. Significance was determined with the Student’s t test or one-way Anova/Tukey test in case values were considered to be normally distributed. If scores served as a parameter, Kruskal-Wallis test was used to determine statistical significance. Differences were considered statistically significant with p≤0.05 and highly significant with p≤0.01.

## Results

### Menstrual-like Bleeding Induced in the Mouse Uterus can be Quantified

**Figure 2:**
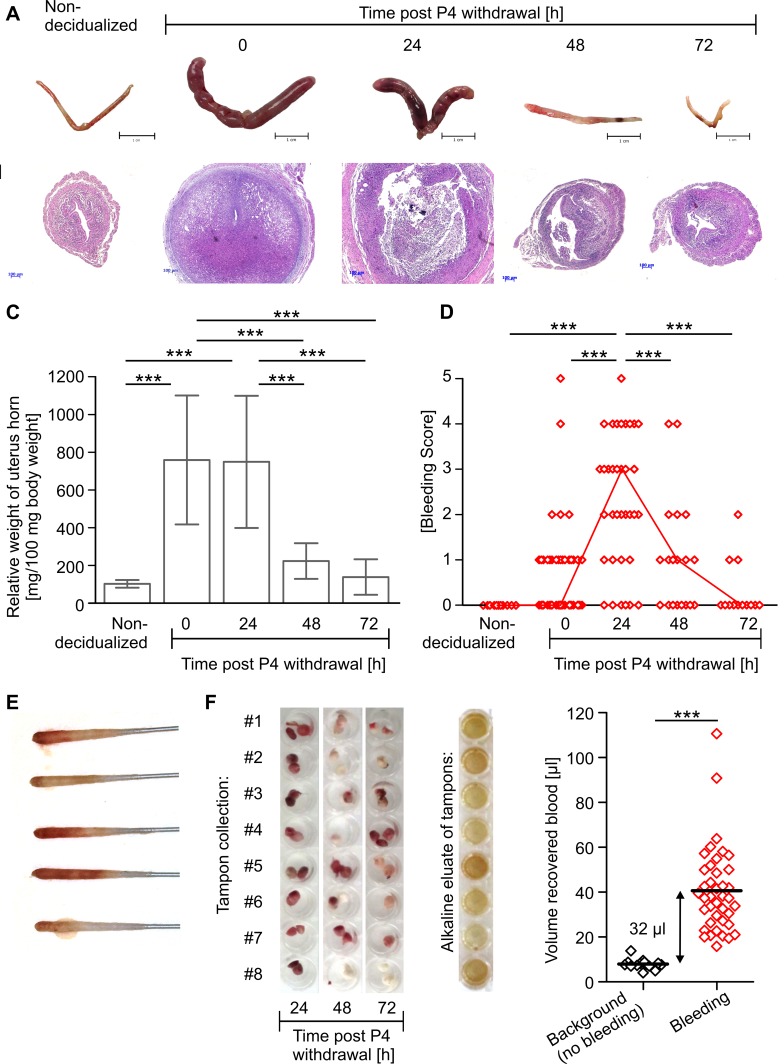
Induction of visible and quantifiable menstrual-like bleeding and associated morphological changes in the mouse uterus. A: Pictures of uteri isolated at indicated points of time. B: Representative H&E stained cross sections of uterus samples. C: Graph shows mean ± SD of relative weight of decidualized uterus horns. D: Bleeding intensity assessed by scoring of vaginal smears is depicted (open red symbols  =  data from individual mouse; red line  =  median). Quantification of uterine blood loss by E: vaginal smears performed 24 h post P4 withdrawal or F: by tampon collection, alkaline hematin elution and photometric measurement. Pooled data from 3 independent experiments are shown (n = 10−40). Significance tested by one-way-Anova/Tukey test (C), Kruskal-Wallis test (D) and Students t test (E) (* p≤0.05, ** p≤0.01, *** p≤0.001).

In order to investigate mechanisms and pathogenesis of uterine bleeding, we adopted a mouse model of hormonal sensitization and decidualization (Figure 1A) [6]–[8]. As reported by others, injection of oil into the uterus of ovariectomized mice sensitized by estrogen (E2) and P4 treatment induces decidualization of the endometrial stroma, which is reflected by a massive expansion of the uterus and an increase in uterine wet weight reaching a maximum between 0 - 24 h after P4 withdrawal and declining thereafter (Figure 2A–C). In addition, histological examination confirms menstrual-like events in the mouse uterus: after intra-uterine oil injection and before P4 withdrawal decidual tissue is present in the uterus as indicated by expansion of stroma, closure of the uterus lumen and thinning of the myometrium. In rare cases, first signs of tissue disintegration and bleeding can be found. 24 h after removal of the P4 releasing implant, massive tissue degradation takes place, i.e. dissociation of decidualized endometrium from basalis and myometrium, increasing interstitial spaces, isolated tissue debris and strong erythrocyte infiltration. Concomitant with loss of tissue integrity, formation of new luminal epithelium can be observed proceeding steadily until 72 h after P4 removal when most uteri are completely reepithelialized, repaired and represent a normal, non-decidualized morphology (Figure 2B).

Importantly, we here show that the described morphological events are associated with menstrual-like bleeding that can be visualized and characterized by vaginal smears (Figure 2E). Evaluation of H/E stained vaginal smears reveals that overt bleeding starts as early as 8 h after P4 removal (data not shown) and is most intense 24 h thereafter, concomitant with strongest signs of tissue degradation. During the repair phase (48–72 h post P4 withdrawal) bleeding intensity decreases and only sporadic and little bleeding is observed 72 h after P4 removal (Figure 2D).

To address the total quantity of bleeding in mice, we established a method comparable to that used to measure menstrual blood loss in women in the clinic [11]. Menstrual-like discharge was collected by tampon-like cotton pads, from which heme chromogens were eluted and quantified by photometric measurement. Using this method we measured a mean total blood loss of 40 µl. In mice, which did not show uterine bleeding, urine and vaginal fluid soaked into the tampons resulted in a mean background equivalent to 8 µl blood, yielding a corrected blood loss of 32 µl across the total bleeding period (Figure 2F).

Thus, we here show that in the described mouse model of menstruation morphological changes of the uterus are associated with visible uterine bleeding. We significantly develop the model further by providing a technical basis allowing the quantification of clinically relevant parameters such as bleeding intensity and total blood loss.

### Menstrual-like Bleeding in Mice Reflects Major Molecular Characteristics of Menstruation in Women

**Figure 3:**
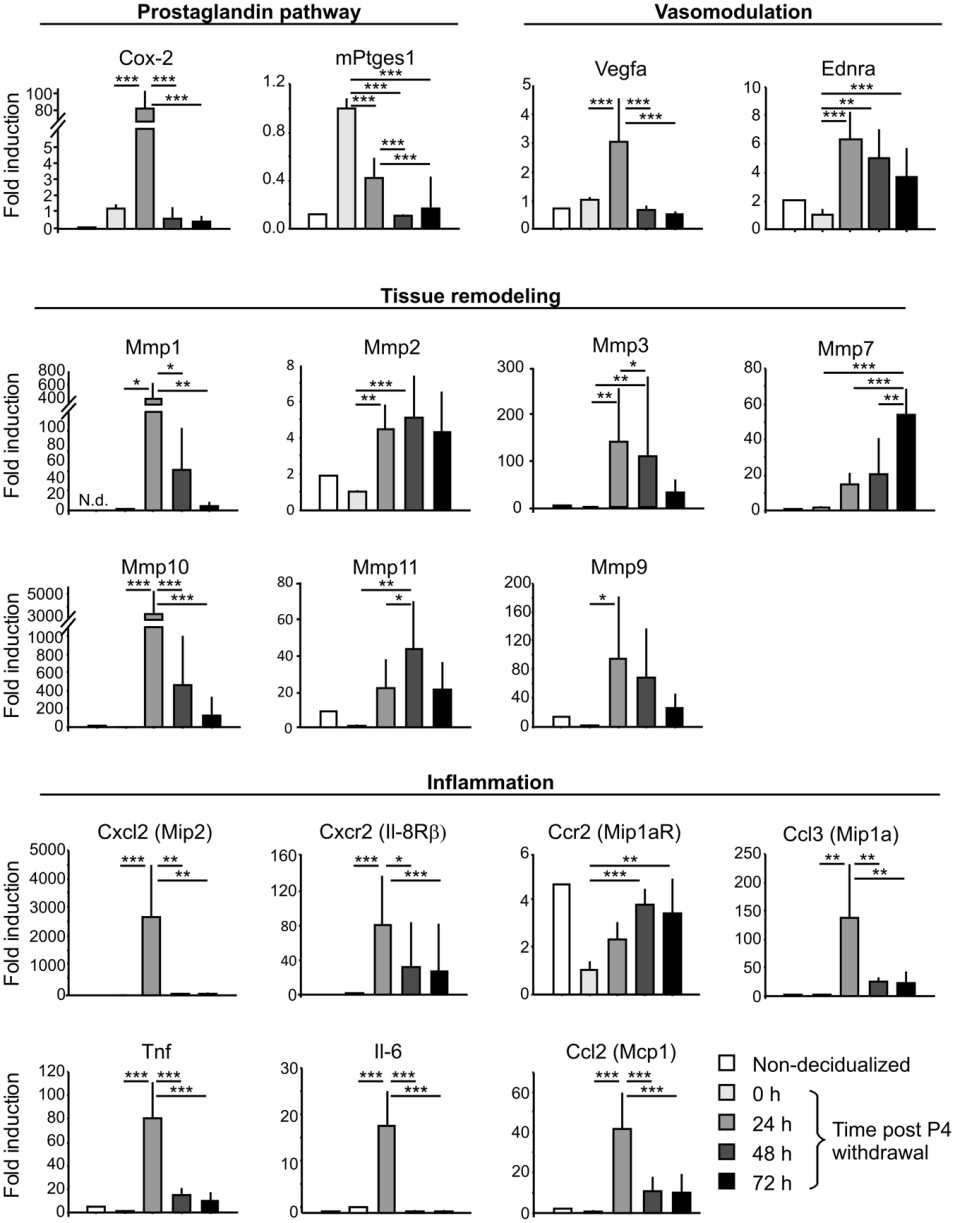
Analysis of molecular events during menstrual-like bleeding in the mouse uterus. Diagrams depict mRNA levels of indicated genes normalized to Cyclophilin A mRNA levels in uterine tissue as analyzed by RT-PCR. One out of 4 independent experiments is shown (n = 6–10 per experiment). Significance tested by one-way Anova/Tukey test (* p≤0.05, ** p≤0.01, *** p≤0.001).

The onset of menstruation in women is characterized by synthesis of prostaglandins, vasoactive and inflammatory mediators, extracellular matrix-modifying enzymes and leukocyte influx [1]. To verify that these characteristics are reflected in the mouse P4 withdrawal model, we performed RT-PCR in uterine tissue (Figure 3). In fact, a variety of genes involved in the production of prostaglandins, e.g. Cyclooxygenase-2 (Cox-2) and prostaglandin E synthase (mPtges1) are strongly upregulated in the uterus in response to decidualization and P4 withdrawal. Intense uterine bleeding is also associated with high expression of vasomodulatory factors, e.g. Vascular endothelial growth factor-A (VEGF-A) and Endothelin receptors (ENDR). In consistence with tissue remodeling events, expression of matrix metalloproteinases (MMPs) is induced upon removal of P4, however, a differential expression pattern across the bleeding phase can be noted: while strong transcription of MMP-1â, -3, -9, and -10 is found at the time of intense bleeding and tissue destruction (24 h post P4 withdrawal), highest transcription of MMP-2, -7 and -11 occurs at later phases (48 and 72 h post P4 withdrawal) concomitant with repair and reepithelialization. Importantly, a wide range of inflammatory mediators including interleukins, chemokines and chemokine receptors are highly and selectively expressed early after P4 withdrawal, indicating leukocyte influx and inflammation in the uterus during the initiation of bleeding.

Therefore, we here confirm that major characteristics and molecular mechanisms of menstruation in women are mimicked by the mouse P4 withdrawal model. Strikingly, we show that onset of bleeding correlates with a strong and instant upregulation of inflammatory mediators implying an involvement of leukocytes in menstrual-like processes.

### Uterine Bleeding is Associated with Dramatic Influx of Granulocyte into the Uterus

**Figure 4:**
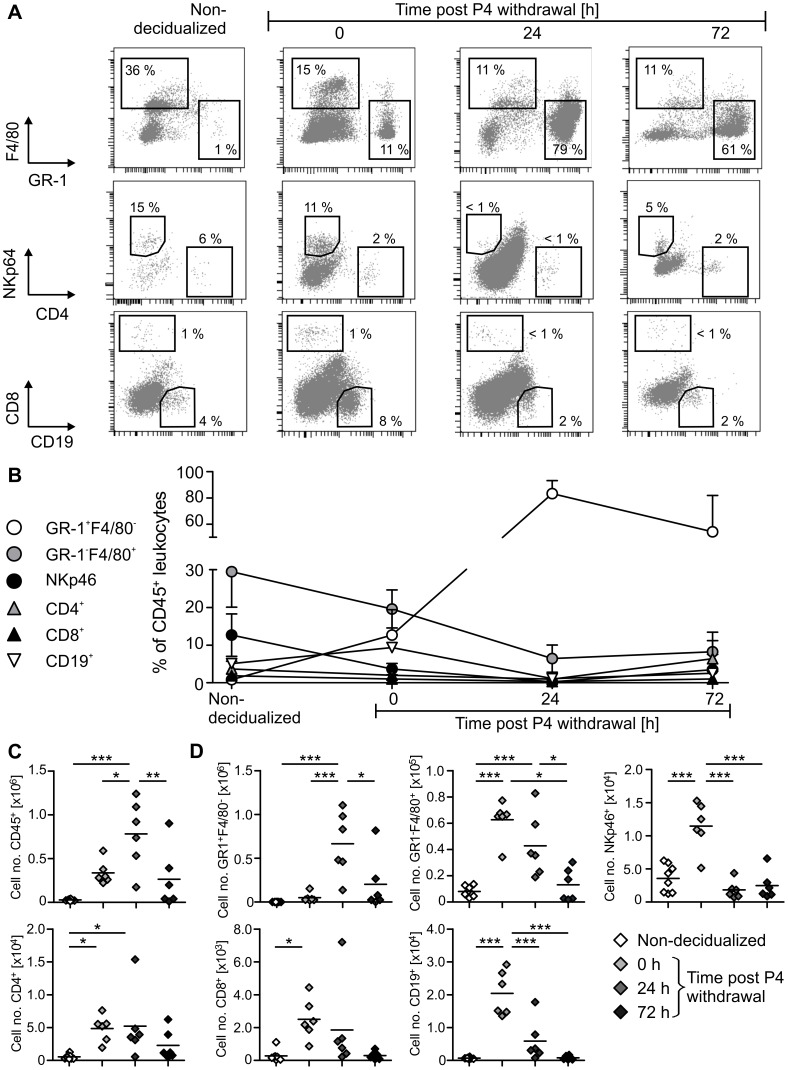
Leukocyte count and composition across induced menstrual-like bleeding in mice. Single cell suspensions were generated from freshly isolated uteri by enzymatic digestion and used for FACS analysis. A: Representative FACS plots gated on live CD45^+^ cells are shown. B: Diagram depicts mean ± SD of frequencies of indicated cell populations among total CD45^+^ leukocytes. C-D: Graphs show total cell number of CD45^+^ leukocytes (C) or of indicated leukocyte subpopulations (D) per uterus horn (symbols  =  data from individual mouse; black line  =  mean). One out of 4 independent experiments is shown (n = 5–6 per experiment). Significance tested by one-way Anova/Tukey test (* p≤0.05, ** p≤0.01, *** p≤0.001).

Since leukocytes are primary mediators of inflammation and play a critical role in many reproductive as well as tissue-remodeling processes, we addressed these cells in the mouse menstruation model by performing flow cytometric analysis of uterine leukocytes (Figure 4).

Prior to decidualization the uterus harbors low, but considerable numbers of CD45^+^ leukocytes including NKp46^+^ NK cells, GR-1^−^F4/80^+^ macrophages and few GR-1^+^F4/80^−^ granulocytes, CD19^+^ B cells, CD4^+^ T helper cells and CD8^+^ cytotoxic T cells (Figure 4B–D). Upon decidualization numbers of CD45^+^ leukocytes dramatically rise, resulting in increased numbers of all immune cell populations investigated here (Figure 4C). During decidualization CD45^+^ leukocytes compose up to 10% of all cells isolated from uterine tissue. Before P4 removal the uterus is mainly populated by macrophages, NK cells and granulocytes, while lymphocytes (i.e. T – and B cells) are present in rather small amounts (Figure 4B/D). Most interestingly, after P4 removal and coinciding with strongest bleeding granulocytes massively infiltrate the uterus and make up 90% of local leukocytes. At the same time, all other investigated cell types strongly decrease in number and frequency (Figure 4B/D). This phenomenon most intensely affects uterine NK cells since NKp46^+^ cells almost completely vanish from the uterus during bleeding. When bleeding ceases and the endometrium is undergoing repair, granulocytes are cleared from the tissue and leukocyte composition approaches patterns similar to those found in the uterus before decidualization (Figure 4B–C).

With these results, we provide a detailed, qualitative and quantitative analysis of all major leukocyte populations in the mouse uterus during induced menstrual-like bleeding. We demonstrate a strong granulocyte influx into the uterus coinciding with the onset of bleeding, while all other immune cells investigated here compose only minor populations.

### Uterine Granulocytes Display Typical Features of Activated Neutrophils and are Critical for Local Tissue-remodeling

**Figure 5:**
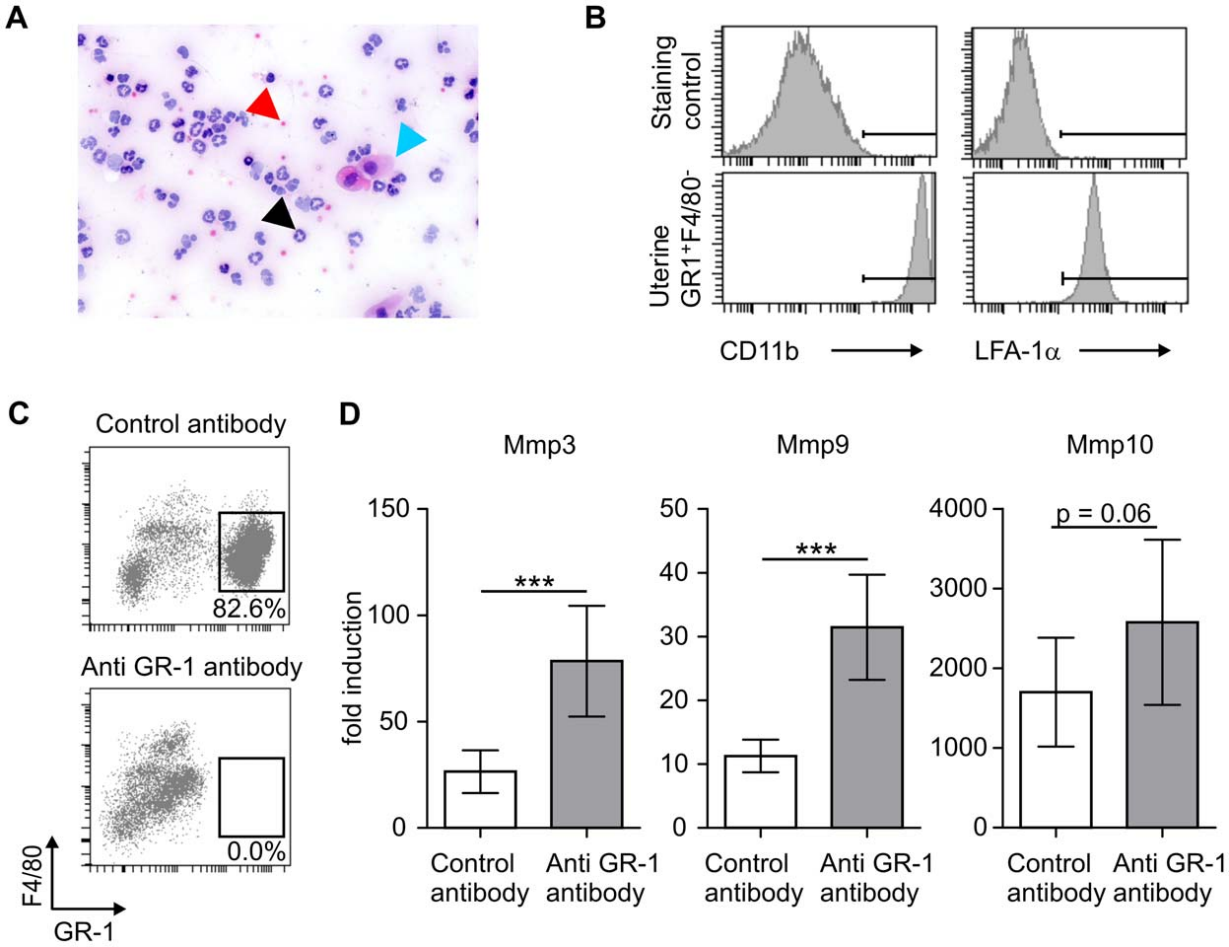
Phenotypic and functional characterization of uterine granulocytes. A: Picture shows H/E stained vaginal smear (24 h post P4 withdrawal). Black arrow indicates polymorph and segmented mononuclear cell, red arrow erythrocyte and blue arrow epithelial cell. B: Expression of CD11â and LFA-1á on uterine CD45^+^GR-1^+^F4/80^−^ cells was assessed by FACS. C-D: Granulocytes were depleted *in vivo* by anti GR-1 antibody. 24 h post P4 withdrawal, depletion was verified by FACS (C). Graphs show relative mRNA levels of MMP3, −9, −10 in the uterus 24 h post P4 withdrawal (D). Data are representative for at least 2 independent experiments (A−B; n = 6 per experiment) and 1 experiment (C−D; n = 9). Significance tested by Students t test (* p≤0.05, ** p≤0.01, *** p≤0.001).

As revealed by H&E staining of vaginal smears, granulocytes present in the uterus during bleeding have segmented and polymorph nuclei and thus, display classical morphological features of neutrophils (Figure 5A). In line with these cellular changes, expression of the major neutrophil chemokine receptor Cxcr2, the homologue of human IL-8â receptor, as well as neutrophil attracting chemokine CxCl2, which represents a mouse homologue of human IL-8 [12], are strongly upregulated 24 h after P4 removal (Figure 3). In addition, uterine granulocytes highly express integrin subunits CD11b (MAC-1) and CD11a (LFA-1á), which are instrumental for transmigration of activated neutrophils into inflamed tissue (Figure 5B) [13].

In order to assess the functional relevance of granulocytes in regulating menstrual-like bleeding, we depleted granulocytes by anti-mouse-GR-1-antibody. Although depletion of GR-1^+^ cells was almost complete as confirmed by FACS analysis in uterine tissue (Figure 5C), no significant change in blood loss via the endometrium was detected (data not shown). However, expression of a variety of tissue-remodeling enzymes, in particular MMP-3 and -9 was considerably increased upon granulocyte depletion (Figure 5D).

Taken together, these data suggest a critical, so far poorly defined role of neutrophils in the regulation of inflammation and tissue remodeling in the uterus during menstrual bleeding.

### Menstrual-like Bleeding is Abrogated by Inhibition of Angiogenesis in Decidualized Endometrium

**Figure 6:**
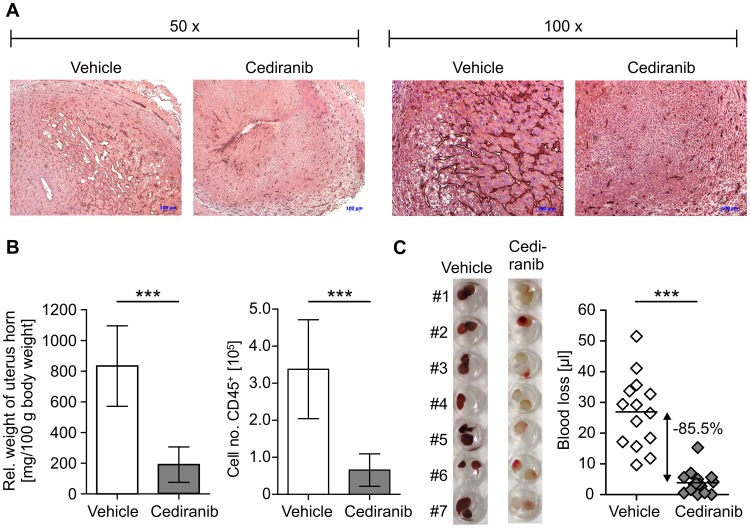
Abrogation of menstrual-like bleeding by inhibition of angiogenesis. Mice were treated with Cediranib (10 mg/kg/d, d 8–15) or received vehicle alone as a control. A: Pictures show immunohistochemical staining (brown) for CD31/PECAM in decidualized uterus samples. B: Relative uterus weight and numbers of CD45^+^ leukocytes/uterus horn upon Cediranib treatment are presented. C: Figure depicts uterine blood collected by tampons (left) and total uterine blood loss measured by alkaline elution of tampons corrected by background (right). Data are representative for 1 out of 3 independent experiments (n = 6−14 per experiment). Significance tested by Students t test (* p≤0.05, ** p≤0.01, *** p≤0.001).

Formation of decidualized tissue is associated with high expression of angiogenic factors such as VEGF (Figure 3) and strong vascularization of the endometrium (Figure 6A) [1]. To address the importance of this process for uterine bleeding, we blocked angiogenesis during decidualization by treating mice with Cediranib (AstraZeneca), a potent inhibitor of VEGF receptor tyrosine kinases. As observed by immunhistochemical CD31 staining, formation of vasculature in the decidualized endometrium was reduced by treatment with Cediranib (Figure 6A), which correlates with a significantly reduced uterus weight and decreased entry of CD45^+^ leukocytes into the uterus (Figure 6B). Importantly, inhibition of angiogenesis resulted in a drastic reduction (85.5%) of menstrual-like bleeding compared with the vehicle group (Figure 6C).

These data for the first time demonstrate that the degree of vascularization in decidualized endometrium critically determines uterine blood loss. We furthermore prove that induced menstruation in mice is sensitive to treatment with respective pharmacological compounds.

## Discussion

Heavy menstrual bleeding (HMB) is a common disorder among pre-menopausal women. It not only considerably impairs quality of life, but also poses a major socio-economic burden to public health systems raising a high need to develop effective and reliable medication against HMB [5]. However, development of novel therapeutics is limited by the incomplete knowledge on mechanisms underlying menstrual bleeding and pathogenesis of associated disorders. In addition, although some progress has been made in the development of animal models [6]–[9], these remain poorly characterized and appropriate techniques allowing evaluation of clinically relevant parameters are still not available.

The current study provides a comprehensive characterization of a mouse menstruation model, confirming that aspects of human menstruation are reflected in a surprisingly natural way. We verify that, as in women, removal of P4 in mice leads to disintegration and shedding of decidualized endometrium and subsequent repair [6], [7] being associated with menstrual-like bleeding [14]. Importantly, we show for the first time that menstrual-like bleeding can be quantified with respect to bleeding intensity and total blood loss. Our findings provide a basis to assess uterine blood loss in a preclinical mouse model. Thereby, these data enable investigation of mechanisms regulating menstrual bleeding as well as testing of therapeutics against menstrual disorders in women.

Mean menstrual blood loss in women is between 30–40 ml, while ranging between 80 ml up to 500 ml in HMB subjects [15]. Considering a total blood volume of 5 l, this corresponds to roughly 1% of total blood in healthy women and 2% up to 10% in heavy menstruating individuals. As measured by our “tampon-method” mice on average lose 32 µl blood, which represents approximately 1.5–2% of their total blood given a blood volume of 2–3 ml [16]. Accordingly, bleeding patterns in the described model closely resemble human menstruation in terms of quantity, with a relative blood loss ranging between normal to heavy bleeding.

Using our novel technique, we here show that the mouse P4 withdrawal model of induced menstruation is sensitive to therapeutic intervention since uterine blood loss was drastically reduced upon inhibition of decidual angiogenesis by treatment with Cediranib. These data demonstrate that menstrual blood loss depends on vascularization of endometrial tissue and imply inhibitors of angiogenesis for the treatment of HMB. In fact, being primarily used to fight cancer, anti-angiogenic drugs have recently been shown to be effective and safe in the treatment of age-related macula degeneration and other indications [17], [18]. Based on our data, local application of anti-angiogenic compounds in the uterus can be regarded as a very effective therapy option in HMB subjects, who preclude pregnancy by contraceptives. However, inhibition of angiogenesis might also interfere with endometrial repair mechanisms as it has been shown that treatment with VEGFtrap markedly delays reepithelialization and vascularization of the regenerating upper endometrium in mice [19]. Nevertheless, our findings clearly identify vascularization of decidualized tissue as a critical determinant of menstrual blood loss.

In addition, our data demonstrate a significant involvement of inflammation and leukocytes in menstrual-like bleeding. It is known that the uterus is populated by a plethora of different leukocyte populations changing in terms of composition and number across the menstrual cycle [2], [20]. While uterine natural killer (uNK) cells occur in high amounts during the implantation window, there is a marked increase in numbers of local macrophages and neutrophils prior to menstruation [20]. Similarly, an increase in endometrial macrophages and neutrophils in the decidualized mouse uterus has been reported previously [21], [22]. In those studies singular markers were used for identifying cells and only few tissue sections were analyzed by immunohistochemistry, which are not necessarily representative for the whole organ. Our study for the first time provides a detailed, qualitative and quantitative analysis of a broad spectrum of leukocyte populations in the mouse uterus across different phases of menstrual-like bleeding. By using a defined combination of surface markers and multi-parameter flow cytometry, we are able to differentiate all relevant leukocyte and lymphocyte populations in parallel. We furthermore offer exact information on relative frequency and cell numbers in the whole uterus horn. We thereby demonstrate that upon decidualization numbers of all immune cells investigated here significantly rise. Strikingly, instantly after P4 removal there is a drastic influx of granulocytes into the uterus, while at the same time other leukocyte populations, in particular uNK cells, decrease in frequency and number. These uterine granulocytes display a typical neutrophil morphology, which is in line with a concomitant induction of neutrophil attracting chemokines and receptors (e.g. Mip2, Cxcr2). In addition, we characterize these cells by showing their high expression of typical activation-associated adhesion molecules CD11á (LFA-1) and CD11â (MAC-1) that allow activated cells to immigrate from the blood stream into inflamed peripheral tissues [13]. Therefore, we extend current knowledge on the involvement of leukocytes in uterine bleeding by demonstrating an immediate and selective influx of activated neutrophils into the uterus on expense of all other immune cell populations analyzed here. Our findings provide further evidence that uterine neutrophils are critical cellular regulators of menstruation.

In line with this hypothesis, neutrophils have the capacity to produce molecular mediators relevant for menstrual bleeding such as prostaglandins (PG) driving vasoconstriction and –dilation of uterine vessels [23]–[25]. These cells secrete significant amounts of IL-8, which is strongly upregulated during menses [26]–[28] and promotes inflammation, angiogenesis and permeability of vascular endothelium [13]. Furthermore, neutrophils are major sources of MMPs and elastase mediating endometrial tissue degradation and reepithelialization [3], [29], [30]. A study by Kaitúu-Lino et al. recently described that neutrophils infiltrating the mouse uterus during induced menstrual-like bleeding specifically express MMP7 and MMP9. In addition, a certain division of labor among MMPs was suggested: while some MMPs (e.g. MMP9) were abundant during tissue breakdown, others (e.g. MMP7) were associated with reepithelialization [29]. In line with these data, we found a differential expression pattern for MMPs, with high expression of MMP9 concomitant with tissue breakdown and delayed upregulation of MMP7 during the repair phase. In this context, it has to be considered that MMP expression levels do not necessarily correlate with MMP protein or activity, which depends on post-transcriptional modification, activation as well as on the presence of their inhibitors (TIMPs). In addition, cellular localization and distribution seem to be critical for MMP activity and their specific function during uterine bleeding in mice [29].

In fact, neutrophils and their effector molecules are of functional relevance in the investigated mouse menstruation model since depletion of these cells has been described to result in delayed reepithelialization and diminished angiogenesis of the regenerating endometrium [21], [31]. Our data complement those studies by demonstrating a significantly increased expression of MMP-3, -9 and -10 in the uterus upon granulocyte depletion. Taken together, our data and previous studies [21], [29], [31] strongly suggest that uterine neutrophils critically determine local tissue breakdown and repair as well as angiogenesis by coordinating expression of matrix-modifying enzymes.

Against these collective findings, it is tempting to speculate that leukocytes could represent a cellular tool to translate hormonal signals into remodeling of the endometrium across the menstrual cycle. However, since leukocytes do not express steroid hormone receptors themselves [20], an indirect hormonal control via cytokines and lipid mediators is likely and requires further research in the future.

Our data show that overt uterine bleeding reflecting many features of human menstruation can be induced in mice. We here identify endometrial vascularization and local granulocyte populations as critical regulators of uterine bleeding and tissue remodeling, respectively. Our data furthermore provide a scientific and technical basis that allows quantification of clinical parameters and prove sensitivity of the model to therapeutic intervention. Thus, the investigated model has great potential for future use in drug discovery and basic research.
